# Initial Experience With VELYS Robot-Assisted Total Knee Replacement: Coronal Plane Accuracy and Effect of Robotic Training on Outcomes

**DOI:** 10.7759/cureus.76323

**Published:** 2024-12-24

**Authors:** Anurag Daxini, Unmesh Mahajan

**Affiliations:** 1 Orthopaedic Surgery, Mahajan Ortho and Surgical Hospital, Nagpur, IND

**Keywords:** better outcomes, robot assisted total knee arthroplasty, robotic knee replacement, total knee replacement (tkr), velys

## Abstract

Introduction: The use of robots for arthroplasty is gaining momentum in recent times to provide accuracy in bony cuts and alignment. We aimed to study the efficacy of coronal plane correction with a new robotic system (VELYS™ Robotic-Assisted Surgery) and also the effect of the learning curve of robot-assisted total knee arthroplasty (RATKA) on outcomes. We hypothesize that the benefits of RATKA are not limited to only surgeons having specific training in robotic knee replacement.

Materials and Methods: A total of 101 RATKAs were performed between November 1, 2022, and December 1, 2022, by a surgeon and all the cases were included in this study. The first 50 consecutive knees were considered as ‘Cohort I’ and the next 51 consecutive knees as ‘Cohort II’. The intraoperative robotic registration data and tourniquet time were recorded. On three months follow‑up, Oxford Knee Score and lower limb scannogram were recorded.

Results: All the 101 cases achieved the desired coronal plane alignment within 3 degrees from neutral. There was a significant difference in the tourniquet time between the two groups. There was no significant difference in the mean three months post-operative values of coronal and sagittal deformity correction, range of flexion, and Oxford Knee Score between the two groups.

Conclusion: The VELYS™​​​​​​​ robot-assisted system produces an accurate correction of coronal alignment. As the surgeon’s experience increases with the system, there is a reduction in tourniquet time; however, the degree of deformity correction is comparable to that when he had no experience. Hence the benefits of RATKA are not limited to only surgeons having specific training in robotic-assisted knee replacement.

## Introduction

The term ‘robot’ was first used in 1921 by Karel Capek in his famous play *Rossum’s Universal Robots* (https://en.wikipedia.org/wiki/Robot#cite_note-KapekWebsite-6). With the advancement in science, robotics has evolved to perform pre-programmed, precise, and repetitive procedures. The first robotic surgical procedure was performed by Kwoh in 1988 using the programmable universal manipulation arm (PUMA) 560 robotic system to undertake neurosurgical biopsies with improved precision [1]. In the field of arthroplasty, the use of robotic technology has enhanced precision by improving implant alignment, accuracy by reducing human error, and outcomes in terms of better post‑operative recovery [2-4].

Studies have shown that the mechanical axis alignment in the coronal plane within a range of 3 degrees from neutral is associated with improved long-term function and increased survival rates [5-10]. In comparison to conventional total knee arthroplasty (TKA), several studies have reported that robot-assisted total knee arthroplasty (RATKA) improved the placement of the femoral component in the antero-posterior plane when compared to the pre-operative plan [11-13] and a more neutral post-operative coronal plane knee alignment [12].

A new robotic system - VELYS™ Robotic-Assisted Solution (VRAS) (DePuy Synthes, Warsaw, IN, USA) - was launched in India in November 2022. This is an imageless robotic system and uses new technology to precisely collect the bony anatomy of the knee. With this information, the surgeon can plan the anatomical placement of the components intraoperatively while preserving the soft tissues. The robotic-assisted saw delivers precise, accurate, and efficient delivery of this implantation plan.

We aimed to study the efficacy of coronal plane correction with this new robotic system and hypothesize that the benefits of RATKA are not limited to only surgeons having specific training in robotic knee replacement.

## Materials and methods

**Figure 1:**
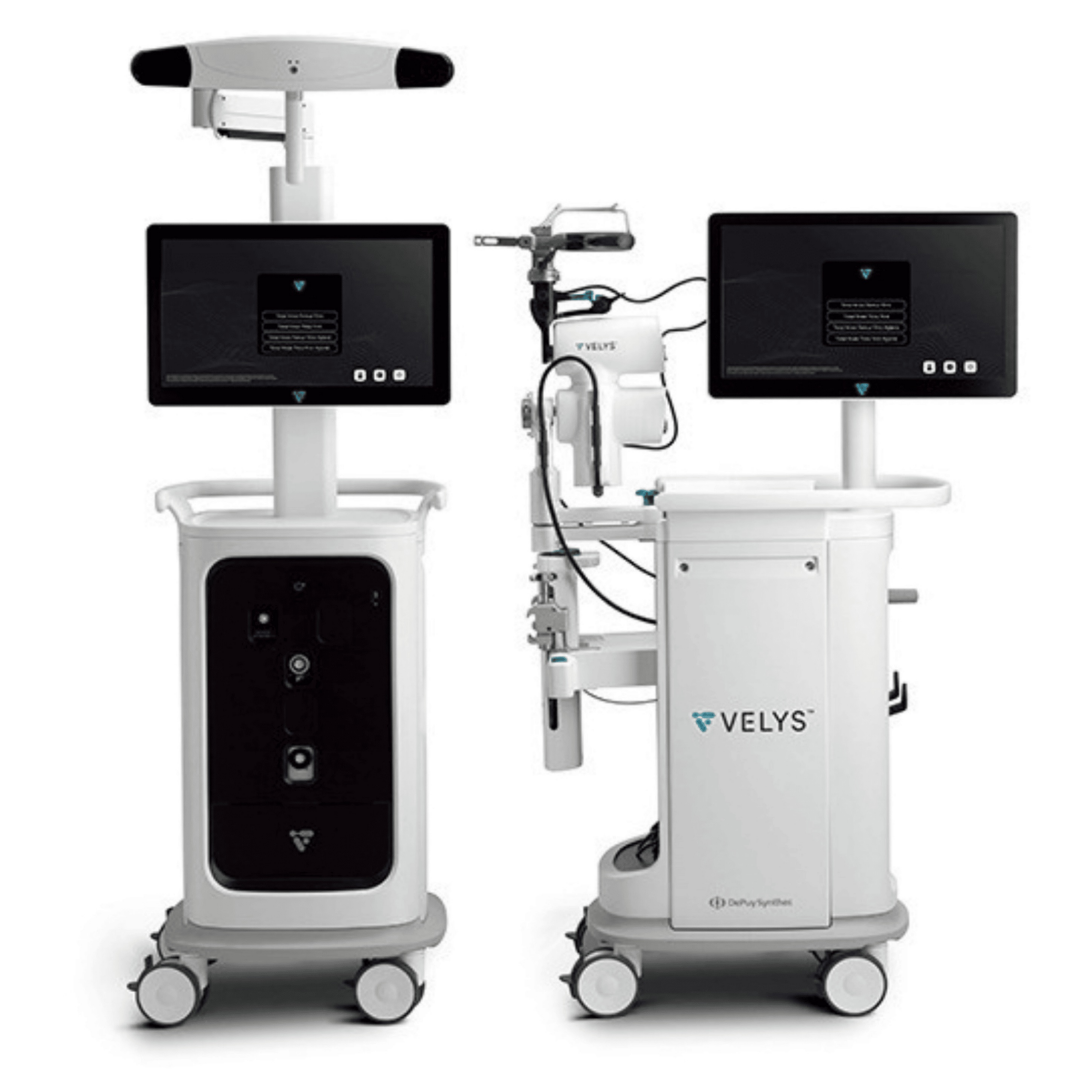
VELYS Robot-Assisted Solution provided by DePuy Synthes

This was a single-center study done on the knees of 101 consecutive patients who underwent RATKA since the time of its inception at our institute by a surgeon and all the patients were included in the study. A total of 101 total knee replacements were performed between November 1, 2022 and December 1, 2022 by a single surgeon using VRAS. Procedures not performed using VRAS were excluded. The study was performed in accordance with the Declaration of Helsinki and was approved by the institutional ethics review board (on June 27, 2022). Informed consent was obtained from all the participants. Pre-operative and three months post-operative coronal and sagittal alignment, intraoperative registration data, and tourniquet time were collected for all the cases. The first 50 knees were grouped as ‘Cohort I' and the next 51 cases were grouped as ‘Cohort II’. All patients were operated by a standard midline incision and medial parapatellar approach. All patients received posterior stabilized knee implants (Attune knee, DePuy Synthes, Warsaw, IN, USA). Patella was resurfaced in all cases. After the final implantation, the tourniquet was deflated and time noted for each case. The patients were followed up postoperatively at six weeks, three months and six months. A follow-up Oxford Knee Score was calculated at three months and a lower limb scannogram was taken to calculate coronal alignment as per the technique described by Babazadeh et al. [14] by an arthroplasty-trained fellow (Figure 1).

Descriptive statistics are reported as means with standard deviations (SD) or medians with interquartile ranges (IQR), based on data distribution. Continuous variables were analyzed using means and SDs and compared with Student's t-test, while categorical variables were expressed as numbers and percentages and analyzed using the chi-square test. A p-value<0.05 was considered statistically significant. Analyses were performed using SPSS software (version 20.0, IBM Corp., Armonk, NY, USA) and Microsoft Excel (2019) (Microsoft Corp, Redmond, WA, USA).

## Results

A total of 101 operated knees consisted of 40 bilateral and 21 unilateral knees (61 patients). There was no statistically significant difference between Cohorts I and II in terms of age, sex, BMI, and laterality as shown in Table 1.

**Table 1:** Demographic data of study showing no statistically significant difference between the two groups in terms of age of patients, BMI, sex and laterality. Abbreviations: BMI, body mass index; NS, not significant. ^a^Student's t-test, ^b^chi-square test.

Parameters	Cohort I	Cohort II	Chi-square / t-Value	p-value
No. of cases	50	51		
Age (years), mean±SD (range)^a^	67.94±7.08 (57.00–86.00)	67.00±7.36 (55.00–87.00)	0.654	0.514 (NS)
BMI (kg/m^2^), mean±SD (Range)^a^	28.07±3.04 (23.00–37.50)	27.14±4.24 (16.70–39.00)	1.269	0.207 (NS)
Sex, n (%)^b^			0.561	0.454 (NS)
Male	14 (28.0)	11 (21.6)		
Female	36 (72.0)	40 (78.4)		
Laterality, n (%)^b^			0.247	0.619 (NS)
Right	24 (48.0)	27 (52.9)		
Left	26 (52.0)	24 (47.1)		

The mean pre-operative varus in Cohorts I and II was 7.88 ±5.66 degrees and 10.86 ±4.95 degrees, respectively. At three months, the mean varus calculated by lower limb scannogram for Cohorts I and II was 0.97 ±0.80 and 0.98 ±0.75 degrees, respectively. There was no statistical difference between the two groups in terms of final post-operative varus achieved (p-value: 0.96) (Table 2).

**Table 2:** Comparison of mean preoperative varus deformity and mean varus at three-month post-operative period. Data shows that the varus correction achieved in both the groups is comparable and not statistically significant. t-Value calculated by Student' t-test; NS=not significant.

Duration	Mean Varus (Mean ± SD)		t-Value	p-value
	Cohort I (N=50)	Cohort II (N=51)		
Pre-op	7.88 ± 5.66	10.86 ± 4.95	0.065	0.96 (NS)
3 months post-op	0.97 ± 0.80	0.98 ± 0.75		

Similarly, the mean pre-operative fixed flexion deformity in Cohorts I and II was 6.54±3.87 degrees and 7.39±6.22 degrees, respectively, while the three-month post-operative fixed flexion deformity was 3.86 ± 2.03 degrees and 3.97±2.71 degrees, respectively. The p-value was 0.85 (non‑significant). 

The mean preoperative range of flexion in Cohorts I and II was 127.38±9.03 degrees and 129.64±5.73 degrees, respectively, and mean three months post-operative flexion was 126.42±7.19 degrees and 126.68±5.63 degrees, respectively. This difference was statistically not significant between the two groups (p-value: 0.84).

The mean tourniquet time in Cohort I was 49.66±8.47 minutes and in Cohort II was 43.06±6.75 minutes, which was statistically significant as shown in Table 3.

**Table 3:** Comparison of mean tourniquet time between the two groups Cohort II has significantly less tourniquet time than Cohort I. t-Value calculated by Student's t-test; *Significant.

Groups	Mean Tourniquet Time (min) (Mean ± SD)
Cohort I (N=50)	49.66±8.47
Cohort II (N=51)	43.06±6.75
Mean difference	6.60
t-Value	4.325
p-Value	*0.000036

The mean pre-operative Oxford Knee Score (OKS) in Cohorts I and II was 20.04±6.26 and 17.14 ±7.75, respectively. The three-month post-operative mean OKS in Cohort I and II was 37.86 ±6.92, and 36.76 ±7.37, respectively. The mean difference between the post-operative and pre-operative values of OKS was not statistically significant (p-value=0.561).

## Discussion

This is the first reported long series of total knee replacement with VRAS. The short‑term results showed that it produces desirable results in terms of coronal plane alignment. The results of VRAS were found to be similar to other robotic TKA studies [15-18].

**Figure 2:**
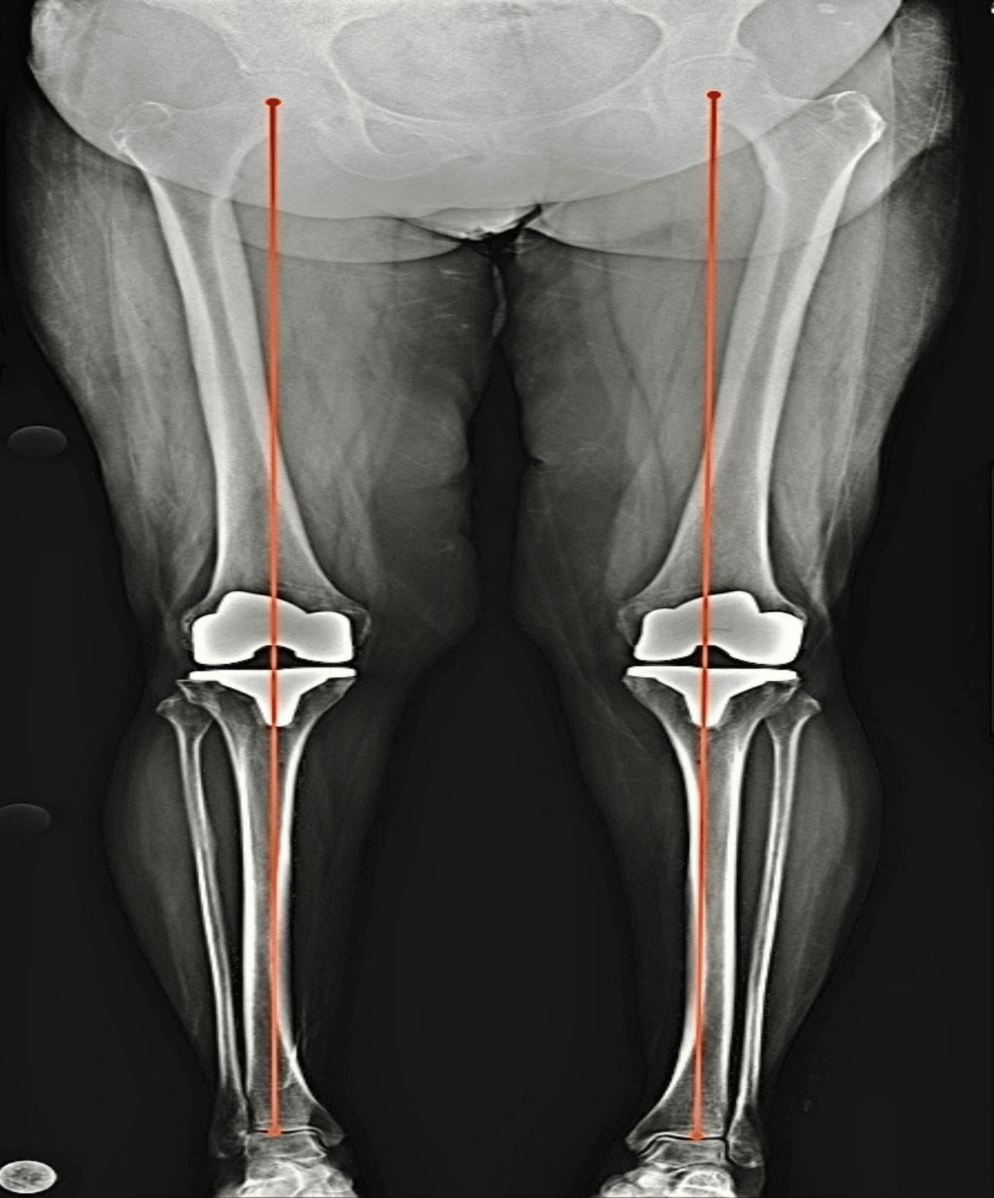
Three-month post-operative scannogram showing mechanical axis of both lower limbs.

Kade Collins et al. evaluated 72 cases of RATKA and achieved the desired coronal alignment of within 3 degrees from neutral in 93.3 % cases [17]. Marchand et al. evaluated 330 RATKAs and achieved accurate coronal correction to within 3 degrees of neutral in 85.7% of the cases [18]. Two studies showed no cases in which the post-operative alignment error was in excess of 1 degree in all the three planes [2,15]. In our study of 101 cases, the desired coronal alignment was achieved in 100% of cases as per the calculation done at three months follow-up using the lower limb scannogram (Figure 2).

The overall mean tourniquet time in our study was 46.3 minutes. It is a well-known fact that the more the surgical time, the more the chances of infection. Olivecrona et al. showed that tourniquet time over 100 minutes increases the risk of complications after knee arthroplasty surgery and special attention is advocated to reduce the tourniquet time [19]. Willis-Owen et al. did a large prospective single-unit study and showed a statistically significant correlation of prolonged operating time and wound infection, with longer operations having a higher incidence of infection. This finding was independent of other variables [20]. In a retrospective review, Peersman et al. [21] showed a link between operating time and infection. In a registry-based study, Ridgeway et al. [22] observed similar findings. We had two cases of post-operative effusion, both presenting three weeks post-surgery. Blood investigations were done, which showed no signs of active infection. Knee aspiration was done and 40 ml and 50 ml of fluid was aspirated in them, respectively. Synovial fluid analysis showed no active signs of infection and since then, there was no recurrence of effusion at six months follow-up. As per Niki et al., it was labelled as nonspecific synovitis from five different types of inflammatory arthritis [23].

In this study, we categorized cases into two groups. The first cohort was included from the time the surgeon adopted this technology. Initially, more tourniquet time was required as the surgical team was not well versed with the system and workflow in terms of the position of personnel and steps of surgery. The tourniquet time was significantly reduced in the second cohort group as the team became well-versed with the system. The other characteristics such as correction of coronal and sagittal deformity were not affected between the two groups, denoting that even the newly adapted surgeons can achieve the desired results with this VAS system as we achieved initially in the first 50 cases.

This study has a few limitations, including limited sample size and short-term follow-up.

## Conclusions

The VELYS™ Robotic-Assisted Solution produces accurate coronal alignment in TKA within 3 degrees of neutral in all the cases. Our study suggests that this RAS system can be easily adopted, safe, and accurate. Also, there is a significant reduction in the operative time with experience. However, the results in terms of deformity correction are comparable. Hence, the benefits of RATKA are not only limited to surgeons having specific training and experience in robotic knee replacement, general orthopedic surgeons should be encouraged to use this innovative technology.
